# ﻿*Chironomus* sp. J – an elusive species from the *Chironomus plumosus* (Linnaeus, 1758) sibling-species group (Diptera, Chironomidae)

**DOI:** 10.3897/compcytogen.19.172398

**Published:** 2025-11-20

**Authors:** Veronika V. Golygina

**Affiliations:** 1 The Federal Research Center Institute of Cytology and Genetics of Siberian Branch of the Russian Academy of Science, Prospect Academika Lavrentieva 10, Novosibirsk, 630090, Russia Russian Academy of Science Novosibirsk Russia

**Keywords:** Banding sequence, *Ch. plumosus* group, evolution, inversion, karyological analysis, karyotype, polytene chromosome, sibling species, species divergence

## Abstract

Data on chromosomal polymorphism in two natural populations from the Inya River in Western Siberia (Novosibirsk province) of *Chironomus* sp. J (Kiknadze, 1991) —one of the sibling species from the *Chironomus plumosus* group — are presented for the first time. The species belongs to the “thummi” cytocomplex with 2n = 8 and the arm’s combination AB CD EF G and is closely related to *Ch. nudiventris* Ryser, Scholl et Wülker, 1983, which has 2n = 6 with the arm’s combination AB CD GEF (a modified “thummi” cytocomplex). The main difference between these two species is the number of chromosomes, apart from that they only differ by the frequencies of banding sequences in arm A, and the presence or absence of some polymorphic inversions. The banding sequence pool of *Chironomus* sp. J consists of 15 banding sequences. Inversions were found in five chromosomal arms – A, B, D, E, F. The most polymorphic arms were B and D. Two studied populations differed by the level of chromosomal polymorphism with one population being completely monomorphic and the other showing high level of polymorphism with 62–65% of heterozygotes and 0.83–0.88 heterozygotic inversion per larva (depending on the year of collection). Comparison of banding sequences to other species from the group showed that *Chironomus* sp. J is indeed closest to *Ch. nudiventris*, with the cytogenetic distance of 0.058 or 0.471 depending on the method of calculation, which indicates that these two species are very closely related. The relationship between *Chironomus* sp. J and other species from the *Ch. plumosus* group was discussed.

## ﻿Introduction

The species *Chironomus* sp. J was first described in the work of I. Kiknadze with coauthors (Kiknadze et al. 1991) based on the larvae collected from the population in the Inya River in Novosibirsk city on the basis of its larvae morphology and karyotype. Although imago of the species has never being studied, it was clear from both its larvae morphology and karyotype that it belongs to the *Chironomus plumosus* (Linnaeus, 1758) group of sibling species. It was indicated that by the karyotype features it is closest to *Chironomus nudiventris* Ryser, Scholl et Wülker, 1983 and differs from it by the number of chromosomes (Kiknadze et al. 1991). Until recently, this was all that was known about this species and in nearly 50 years since its first discovery it has not been found anywhere else, except two populations from the Inya River (Novosibirsk Province, Western Siberia), although during this time hundreds of water bodies from different regions of the Palearctic part of Eurasia have been studied by many researchers. In our previous works we analyzed relations between the main banding sequences of *Chironomus* sp. J and banding sequences of other species from the *Ch. plumosus* group (Golygina and Kiknadze 2008, 2012, 2018) and described the banding sequence pool of the species (Kiknadze et al. 2016), but no information on quantitative data of chromosomal polymorphism, such as frequencies of banding sequences, level of heterozygosity in populations etc., has been published to date.

Here we present data on chromosomal polymorphism in two natural populations from the Inya River in Western Siberia (Novosibirsk province) of *Chironomus* sp. J that are currently known and discuss the relationship of this species with other species from the *Ch. plumosus* group.

## ﻿Material and methods

Specimens from two natural populations from the Inya River (Novosibirsk province, Western Siberia, Russia) of *Chironomus* sp. J were studied (Table 1). The population NSK-IN is located about two km from the mouth of the Inya River, while NSK-IT is located approximately 100 km upstream from NSK-IN. The width of the river at the NSK-IN site was about 100 m, and at the NSK-IT site, about 60 m. At NSK-IN, larvae were collected 4–6 m from the river bank from gray silt, and at NSK-IT in 2001, larvae were collected from dark-gray silt in the middle of the river. The IV instar larvae from the population NSK-IT collected by the author in 2001 were used for polytene chromosome’s slide preparation (Table 1). The age of larvae was determined by the stage of development of the imaginal discs on the first and second segments (Wülker and Gütz 1968). The larvae were fixed with a mixture of 96% ethanol and glacial acetic acid (3:1 *v/v*) and stored at –20 °C. Squashes of polytene chromosome from salivary glands were prepared by a routine aceto-orcein method (Keyl and Keyl 1959; Kiknadze et al. 1991). In addition, permanent slides of polytene chromosomes from the collection of the Institute of Cytology and Genetics of the Siberian Branch of the Russian Academy of Science (Novosibirsk, Russia) were used for the analysis of chromosomal polymorphism in the NSK-NI and NSK-IT populations collected in 1986–1988 (Table 1).

**Table 1.** Collection sites.

Collection locality	Abbreviation	Collection date	Geographic coordinates	Number of larvae
**RUSSIA**				
**Novosibirsk province**				
Inya River in Novosibirsk city	NSK-IN	09.07.1986	54°58'30"N, 83°01'57"E	16
		13.07.1987		12
Inya River in Toguchin town	NSK-IT	02.03.1988	55°14'06"N, 84°24'48"E	3
		08.07.2001		11

Analysis of polytene chromosomes was performed using microscope “Axioskop” 2 Plus at magnifications x20, x40 (overall examination of karyotypes, preliminary identification of banding sequences) and x100 (final identification of banding sequences). Photographs of polytene chromosomes were made at x100 magnification using equipment of the Centre of Microscopical analysis of biological objects SB RAS in the Institute of Cytology and Genetics (Novosibirsk): microscope “Axio Skope.A1”, CCD-camera “Axiocam 512 Color”, software packages AxioVision 4 and Zen (Zeiss, Germany).

Chromosomal mapping of arms A, C, D, E and F was done using mapping system created by Keyl (1962) and Devai et al. (1989), with the karyotype of *Ch. piger* Strenzke, 1959 as the standard one. The mapping of arm B was done according to Maximova (1976) mapping system improved by Schobanov (1994), with the *Ch. plumosus* chromosomes as a standard. Each banding sequence is given the following short designation: a three-letter abbreviation of the species name (spJ), followed by the name of the arm and the serial number of the banding sequence in this arm (according to the order of its discovery), preceded by a letter indicating its geographical distribution in the genus *Chironomus* (p’ for Palearctic sequences or h’ for Holarctic sequences). Thus, for example, h’spJE1 means that while *Chironomus* sp. J itself is a Palearctic species, this banding sequence is identical to banding sequences of some other species and have been found not only in Palearctic, but also in Nearctic populations of these species, thus it has a Holarctic distribution.

Statistical analysis and calculation of cytogenetic distances were performed using the PHYLIP software package (https://phylipweb.github.io/phylip/). Cytogenetic distances were calculated using the Nei method (Nei 1972), each banding sequence was considered as a separate allele.

Calculation of the cytogenetic distance between *Chironomus* sp. J and *Chironomus nudiventris* was conducted using two approaches. The first type of cytogenetic distance – D_cg1_ – was calculated only based on the similarity between the banding sequences in arms E and G, without taking into consideration the fact that arms E and G are joined in *Ch. nudiventris*. Thus, if the banding pattern h’spJE1 is identical to h’nudE1, they were treated as identical, even though the true left arm of chromosome III of *Ch. nudiventris* has additional bands that come from arm G, and different inversions may be present in this part of the arm. The second type of cytogenetic distance – D_cg2_ – was calculated by the method used in the work of Gunderina and Kiknadze (2000), where banding sequences of left arm of chromosome III (EG) of *Ch. nudiventris* were considered different from the banding sequences of arms E and G of all other species, regardless of the similarity of the banding patterns. The first approach allows us to more accurately estimate the true rate of divergence between banding sequences of compared species, whereas the second approach gives us a more accurate rate of divergence of the karyotypes as a whole, since it takes into account such major event as the fusion of chromosomes.

## ﻿Results and discussion

**Figure 1.**
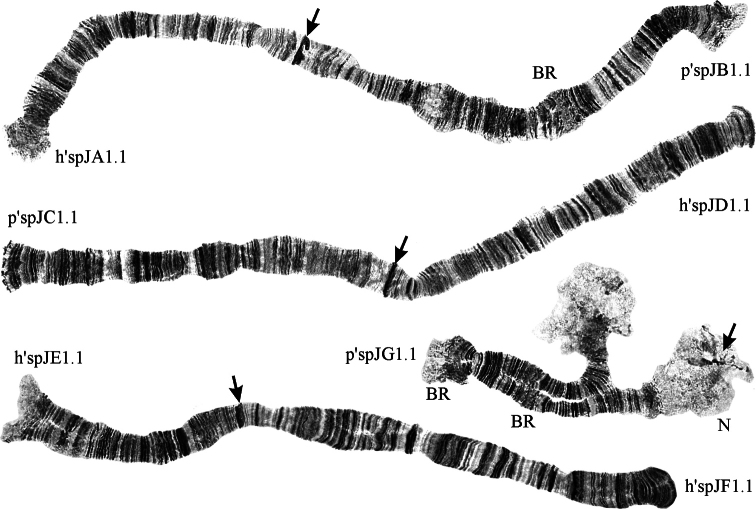
Karyotype of *Chironomus* sp. J; h’spJA1.1, p’spJB1.1 etc. – genotypic combinations of banding sequences; BR – Balbiani Rings, N – nucleolus; arrows indicate centromeric bands.

*Chironomus* sp. J belongs to the “thummi” cytocomplex with a haploid number of chromosomes n = 4 and the arm’s combination AB CD EF G. Chromosomes I (AB) and II (CD) are metacentric, chromosome III (EF) is submetacentric, and chromosome IV (G) is telocentric (Fig. 1). The homologues of arm G are partially unpaired in the region of the terminal nucleolus. The centromeric regions are prominent and easily identifiable. The single nucleolus is located on arm G near the centromere. Two Balbiani Rings (BR) are developed on the arm G, and one BR is located on arm B (Fig. 1). The activity of BR can vary, so all three BR are not always visible.

Currently, the total banding sequence pool of *Chironomus* sp. J consists of 15 banding sequences. Paracentric inversions were found in 5 chromosomal arms – A, B, D, E, F. Arms C and G in both studied populations were monomorphic. Mapping of all banding sequences is presented in Table 2, photographs of the banding sequences in arms A, B, C, E, F were presented in our earlier work (Kiknadze et al. 2016).

**Table 2.** Mapping of banding sequences of *Chironomus* sp. J (regions of inversions are marked in bold).

Designation of banding sequence	Mapping of banding sequence
h’spJA1	1a-2c 10a-12a 13ba 4a-c 2g-d 9e-4d 2h-3i 12cb 13c-19f C
p’spJA2	1a-2c 10a-12a 13ba **13f-c 12bc 3i-2h 4d-9e 2d-g 4c-a** 14a-19f C
p’spJB1	25s-e **21t-16a 25d-23f 15g-r** 22a-23e 15f-12v C
h’spJB2	25s-e 15r-g 23f-25d 16a-23e 15f-12v C
p’spJB3	25s-e 15r-g 23f-k **15a-f 23e-16a 25d-23l** 14r-12v C
p’spJC1	1a-d 11f-d 6gh 17a 2f-3b 2c-1e 11g-15e 8a-11c 6b-3c 6c-f 7a-d 16a-h 2ed 17b-22g C
h’spJD1	1a-2c 15e-16c 18d 8a-10a 13a-12a 18c-a 7g-4a 10e-b 13b-15d 2d-3g 11a-c 16d-17f
	18e-24g C
p’spJD2	1a-2c 15e-16c 18d 8a-d **21a-18e 17f-16d 11c-a 3g-2d 15d-13b 10b-e 4a-7g 18a-c 12a-13a 10a-9a** 21b-24g C
p’spJD3	1a-2c 15e-16c 18d 8a-d 21a-19e **4ba 10e-b 13b-15d 2d-3g 11a-c 16d-17f 18e-19d** 4c-7g 18a-c 12a-13a 10a-9a 21b-24g C
h’spJE1	1a-3e 5a-10b 4h-3f 10c-13g C^†^
	1a-3a 4c-10b 3e-b 4b-3f 10c-13g C^‡^
p’spJE2	1a-2e **11a-10c 3f-4b 3b-e 10b-4c 3a** 11b-13g C^‡^
h’spJF1	1a-d 6e-1e 7a-10b 18ed 17d-11a 18a-c 10dc 19a-23f C
p’spJF2	1a-d 6e-1e 7a-10b 18ed 17dc **15i-17b** 15h-11a 18a-c 10dc 19a-23f C
p’spJF3	1a-d 6e-1e 7a-10b 18ed 17d-15i **11e-15h** 11d-a 18a-c 10dc 19a-23f C
p’spJG1	Not mapped

^†^ - mapped according to Keyl (1962). ^‡^ - revised mapping according to Golygina and Kiknadze (2018).

**Arm A** has 2 banding sequences, both are not unique for the species. The main banding sequence h’spJA1 is identical to h’nudA2, while p’spJA2 is identical to p’nudA1=h’entA4 and differs from h’spJA1 by a simple inversion in the middle of the arm (Table 2). The banding sequence p’spJA2 was found only in the heterozygous state in the population NSK-IN (Tables 3, 4).

**Table 3.** Frequencies of genotypic combinations of banding sequences and general characteristics of chromosomal polymorphism in populations of *Chironomus* sp. J.

Genotypic combination	NSK-IN		NSK-IT	
	09.07.1986	13.07.1987	02.03.1988	08.07.2001
	16^§^	12	3	11
h’spJA1.1	0.937	0.833	1	1
h’spJA1.p’spJA2	0.063	0.167	0	0
p’spJB1.1	0.625	0.587	1	1
p’spJB1.h’spJB2	0.250	0.413	0	0
p’spJB1.3	0.125	0	0	0
p’spJC1.1	1	1	1	1
h’spJD1.1	0.749	0.833	1	1
h’spJD1.p’spJD2	0.188	0.167	0	0
h’spJD1.p’spJD3	0.063	0	0	0
h’spJE1.1	0.937	0.917	1	1
h’spJE1.p’spJE2	0.063	0.083	0	0
h’spJF1.1	0.874	1	1	1
h’spJF1.p’spJF2	0.063	0	0	0
h’spJF1.p’spJF3	0.063	0	0	0
p’spJG1.1	1	1	1	1
% of heterozygous larvae	62.5	66.7	0	0
Number of heterozygous inversions per larvae	0.88	0.83	0	0
Number of banding sequences	15	11	7	7
Number of genotypic combinations of banding sequences	15	11	7	7

^§^ - number of larvae studied.

**Table 4.** Frequencies of banding sequences in populations of *Chironomus* sp. J.

Genotypic combination	NSK-IN		NSK-IT	
	09.07.1986	13.07.1987	02.03.1988	08.07.2001
	16^|^	12	3	11
h’spJA1	0.969	0.917	1	1
p’spJA2	0.031	0.083	0	0
p’spJB1	0.813	0.794	1	1
h’spJB2	0.125	0.206	0	0
p’spJB3	0.062	0	0	0
p’spJC1	1	1	1	1
h’spJD1	0.875	0.917	1	1
p’spJD2	0.094	0.083	0	0
p’spJD3	0.031	0	0	0
h’spJE1	0.969	0.959	1	1
p’spJE2	0.031	0.041	0	0
h’spJF1	0.938	1	1	1
p’spJF2	0.031	0	0	0
p’spJF3	0.031	0	0	0
p’spJG1	1	1	1	1

^|^ - number of larvae studied.

**Arm B** has 3 banding sequences: the main banding sequence p’spJB1 is identical to p’nudB1, the banding sequences h’spJB2 and p’spJB3 are identical to h’nudB3=h’entB1=h’murB1 and p’nudB4, respectively. Both h’spJB2 and p’spJB3 were found only as heterozygotes in the population NSK-IN (Tables 3, 4). Although p’spJB1 is the main banding sequence of this species due to its high frequency, h’spJB2 should be considered as the most ancient of the three banding sequences, since it is the closest to banding sequences of other species of the *Ch. plumosus* group (Golygina and Kiknadze 2008). Both p’spJB1 and p’spJB3 originated from h’spJB2 by simple inversions (Table 2).

**Arm C** is monomorphic. Banding sequence p’spJC1 is identical to p’nudC1.

**Figure 2.**
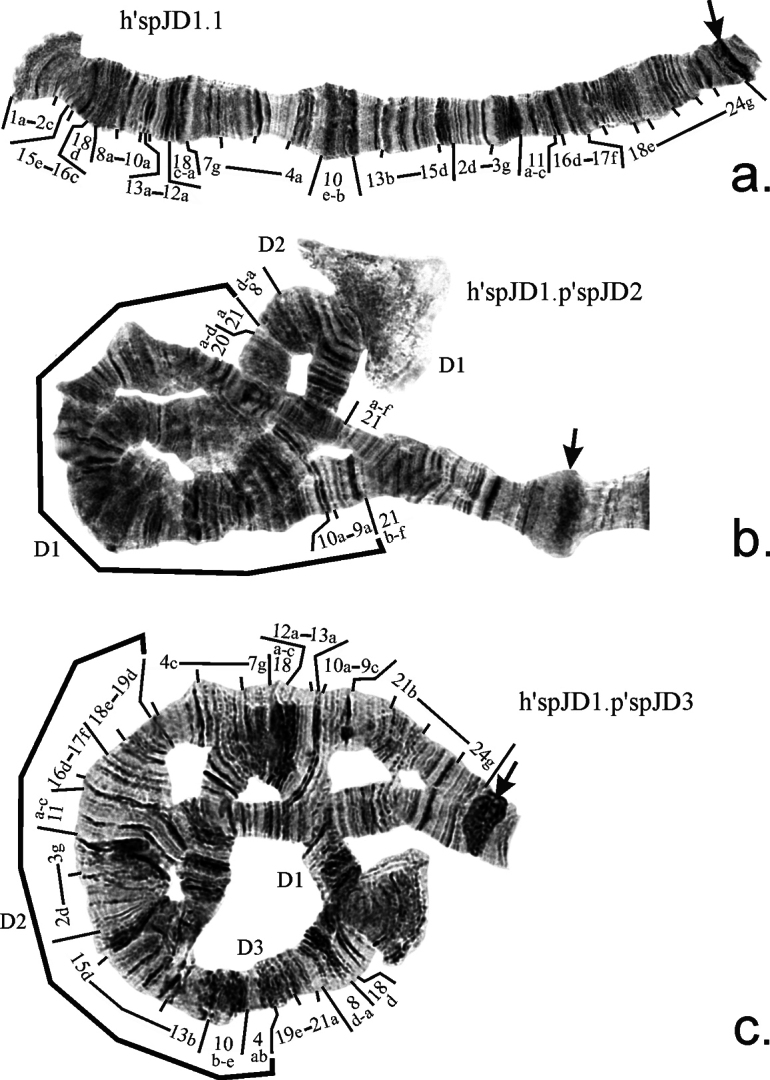
Banding sequences of arm D of *Chironomus* sp. J. Arrows indicate centromeric regions. Brackets show regions of inversions. Regions of chromosomal arms designated by Arabic numerals, small letters indicate separate bands in a region. h’spJD1.1, h’spJD1.p’spJD2 etc. – designations of genotypic combination of banding sequences. D1, D2, D3 – shortened designation of banding sequence (i.e. D1 for h’spJD1), if placed near chromosome, it indicates banding sequence of the particular homologue, if placed above or below a bracket, it indicates banding sequence, from which the homologue derived from.

**Arm D** has 3 banding sequences. The main banding sequence h’spJD1 (Fig. 2a) is identical to h’nudD1=h’entD1, and the banding sequence p’spJD2 (Fig. 2b) is identical to p’nudD2. In our earlier work (Kiknadze et al. 2016) it was assumed that p’spJD2=p’nudD2 originates from h’spJD1=h’nudD1=h’entD1 by complex inversion (one large inversion and one subsequent microinversion on the right border of the first one). However, the analysis of p’spJD2 and reanalysis of p’nudD2 showed that although these banding sequences are indeed identical, they actually differ from h’nudD1 only by one simple inversion, which affects most of the arm, and there is no microinversion in either of them. The position of the right breakpoint of the inversion should also be placed not between bands 21b and 21c, but between bands 21a and 21b (Table 2, Fig. 2b).

The banding sequence p’spJD3 was earlier considered identical to p’nudD4 (Kiknadze et al. 2016). Thus, it was assumed that p’spJD3 differed from p’spJD1 by three inversion steps: one large inversion, one microinversion near its right breakpoint, and on inversion in the middle of the arm. However, after the reanalysis of p’spJD3, it became clear that it was not identical to p’nudD4. In fact, just as p’spJD2, the banding sequence p’spJD3 does not contain a microinversion and differs from p’spJD2=p’nudD2 by a single inversion in the middle of the arm (Fig. 2c). At the same time, p’nudD4 differs from p’spJD3 only by the microinversion of bands 21b 9ab. Thus, p’spJD3 is an intermediate banding sequence between p’nudD2=p’spJD2 and p’nudD4 and currently can be considered species-specific for *Chironomus* sp J. Both p’spJD2 and p’spJD3 were found only in heterozygoous state in the population NSK-IN (Table 3, 4).

**Arm E** has 2 banding sequences: the main banding sequence h’spJE1 is identical to h’nudE1, as well as to the banding sequences of most other species in the group (Golygina and Kiknadze 2018), and p’spJE2 is identical to p’nudE2. The banding sequence p’spJE2 differs from h’spJE1 by a simple inversion (Table 2). It was found as a heterozygote in the population NSK-IN (Table 3, 4).

**Arm F** has 3 banding sequences. The main banding sequence h’spJF1 is identical to h’nudF1=h’pluF1=h’entF1=h’murF1 (Golygina and Kiknadze 2018). The banding sequences p’spJF2 and p’spJF3 differ from it by short simple paracentric inversions (Table 2) and are unique for *Chironomus* sp. J. They both were found only once in a heterozygous state in the population NSK-IN.

**Arm G** is monomorphic. The main banding sequence p’spJG1 is identical to p’nudG1.

The two populations studied differed both in the level of heterozygosity and, correspondingly, in the number of banding sequences present: while the population NSK-IT was monomorphic, the population NSK-IN showed rather high level of inversion polymorphism (Table 3). At present, we can only speculate about factors that can cause such a difference in the level of polymorphism between two studied populations, since there is no data on water quality and sediment profile in the population’s sites. One of the possible reasons for this difference may be possible anthropogenic pollutions in NSK-IN, since this collection site is situated within Novosibirsk city and, moreover, is located downstream from the bridge on the street “Bol’shevistskaya”. This street is the main road for all transport heading south from Novosibirsk, and has very heavy round-the-clock traffic, so we can expect some pollution of river sediments from gas emissions. But it is also probable that if more specimens had been available from NSK-IT, at least some inversions would have been found that would offset the difference between populations that we are currently observing. Although the number of specimens in both populations studied was rather low, the fact that at least in the population NSK-IN, after studying only 28 larvae, 15 banding sequences in 15 genotypic combinations were found indicates that *Chironomus* sp. J is a polymorphic species, and we can expect to see at least an average level of chromosomal polymorphism in its populations.

As can be seen from the results of the banding sequence comparison, the closest species to *Chironomus* sp. J is indeed *Ch. nudiventris*, as previously suggested (Kiknadze et al. 1991). But the analysis of chromosomal polymorphism in *Chironomus* sp. J shown that not only are its main banding sequences identical to those of *Ch. nudiventris*, but even most of the other banding sequences that were found in heterozygous state are also not unique to the species. So, basically, *Chironomus* sp. J and *Ch. nudiventris* differ only by the number of chromosomes and the frequencies of the main banding sequences in arm A – the banding sequence h’spJA1=h’nudA2, which is dominant in *Chironomus* sp. J, is less common in *Ch. nudiventris* (Gunderina and Kiknadze 2000). This proves that these two sibling species are very closely related. Between other species from the *Ch. plumosus* group, *Ch. muratensis* Ryser, Scholl et Wülker, 1983 is also closely related to *Ch. nudiventris* and *Chironomus* sp. J, since it has identical banding sequences in the arms B, E, and F, and the banding sequences in arms A, C and D differ from those of *Chironomus* sp. J by one or two inversion steps. The most prominent difference between *Chironomus* sp. J and *Ch. muratensis* is the banding pattern of the arm G.

We calculated the cytogenetic distance between *Chironomus* sp. J and *Chironomus nudiventris*, using two approaches as described in the “Material and methods” section. The values of D_cg1_ and D_cg2_ between *Chironomus* sp. J and *Ch. nudiventris* were 0.058 and 0.471, respectively. The value of D_cg1_ (0.058) is similar to the distance between such species as *Ch. agilis* Shobanov et Djomin, 1988 and Chironomus sp. prope agilisKiknadze et al. 1991 (0.163), which mainly differ by the size of centromeric heterochromatin, while their banding sequences are mostly identical (Gunderina and Kiknadze 2000). As expected, the second distance D_cg2_ (0.471) is higher than the first, since the arms E and G of *Chironomus* sp. J and the arm EG of *Ch. nudiventris* are considered different. However, it is much lower than the distance between *Ch. plumosus* and *Ch. borokensis* Kerkis, Filippova, Shobanov, Gunderina et Kiknadze, 1988 (0.843) – another closely related pair of sibling species that also have a lot of identical banding sequences and differ mainly by the size of centromeric heterochromatin.

It is necessary to note that the presence in polymorphic stat of banding sequences (such as p’spJA2, h’spJB2, p’spJB3, p’spJD2, p’spJE2) which are identical to polymorphic banding sequences of *Ch. nudiventris* and several other species of the group is a clear indication that all these banding sequences originated before the separation of these species and were present in an ancestral species. At the same time, the presence of the banding sequence p’spJC1 is a clear sign that *Chironomus* sp. J and *Chronomus nudiventris* separated from each other after the separation of other species from this subgroup, such as *Ch. entis* Shobanov, 1989 and *Ch. muratensis*, since this banding sequence was found only in *Ch. nudiventris* and *Chironomus* sp. J (Golygina and Kiknadze 2012). The fact that p’spJD3 is an intermediate banding sequence between p’nudD2 and p’nudD4 (which means that it was present in the banding sequence pool of an ancestor species before the separation of *Chironomus* sp J and *Ch. nudiventris*) raises the question of whether the absence of this banding sequence in *Ch. nudiventris* banding sequence pool is genuine, i.e. a microinversion that differs p’nudD4 from p’spJD3 has been indeed fixated in this species while the banding sequence identical to p’spJD3 has been eliminated over time, or it does exists but we have not yet been able to find it due to its rarity or difficulties in its identification.

Since only two populations with a small number of specimens were examined in *Chironomus* sp. J, it is too early to make any definitive conclusions about the features of its chromosomal polymorphism. However, based on the data we currently have, we can still expect that this species has a significant level of polymorphism, with the arms B and D being more polymorphic than others.

The range of *Chironomus* sp. J is currently limited to about 100 km separating Novosibisk city and Toguchin town and is the smallest among all species from the *Ch. plumosus* group, with the exception of the relatively recently described *Chironomus* sp. K from Japan (Golygina and Ueno 2008). It remains to be clarified later whether *Chironomus* sp. J is endemic of the Inya River or, for example, this species actually prefers riverine habitats, while researchers interested in the genus *Chironomus* mainly collect samples in lakes and ponds, skipping the sites where *Chironomus* sp. J lives, and this is the main reason that for almost 50 years of research we have not been able to find it nowhere else, and it remains as elusive as a unicorn.
